# An overview of the BOIN design and its current extensions for novel early-phase oncology trials

**DOI:** 10.1016/j.conctc.2022.100943

**Published:** 2022-06-13

**Authors:** Revathi Ananthakrishnan, Ruitao Lin, Chunsheng He, Yanping Chen, Daniel Li, Michael LaValley

**Affiliations:** a Bristol-Myers Squibb (BMS), 300 Connell Drive, Berkeley Heights, NJ, 07922, USA; b Department of Biostatistics, The University of Texas MD Anderson Cancer Center, Houston, TX, 77030, USA; c BMS, Seattle, WA, 98109, USA; d Department of Biostatistics, Boston University School of Public Health, Boston, MA, 02118, USA

## Abstract

Bayesian Optimal Interval (BOIN) designs are a class of model-assisted dose-finding designs that can be used in oncology trials to determine the maximum tolerated dose (MTD) of a study drug based on safety or the optimal biological dose (OBD) based on safety and efficacy. BOIN designs provide a complete suite for dose finding in early phase trials, as well as a consistent way to explore different scenarios such as toxicity, efficacy, continuous outcomes, delayed toxicity or efficacy and drug combinations in a unified manner with easy access to software to implement most of these designs. Although built upon Bayesian probability models, BOIN designs are operationally simple in general and have good statistical operating characteristics compared to other dose-finding designs. This review paper describes the original BOIN design and its many extensions, their advantages and limitations, the software used to implement them, and the most suitable situation for use of each of these designs. Published examples of the implementation of BOIN designs are provided in the Appendix.

## Graphical abstract

**Figure:**
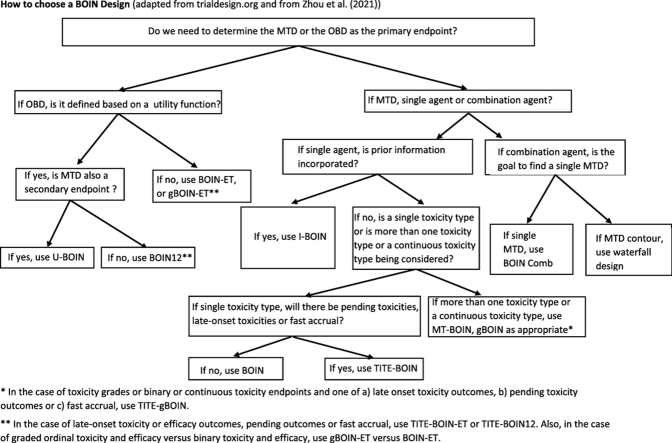

## 1 Introduction

The Bayesian Optimal Interval (BOIN) design and its extensions are a class of early phase dose-finding model-assisted designs used to determine a suitable dose for consideration in later phase oncology trials. The original BOIN design only considered toxicity in determining the maximum tolerated dose (MTD), but the main aim of these extension designs, especially those that consider both efficacy and toxicity in dose finding, is to determine a dose of the study drug that is efficacious but not too toxic. This is called the optimal biological dose (OBD). In chemotherapy drugs, it is assumed in general that there is a monotone increasing dose-response relationship for both toxicity and efficacy. Thus, it is meaningful to find a maximum dose that can be well tolerated by the population, i.e., the MTD. However, in many of the newer immuno-oncology drugs, although the toxicity of the drug increases with an increase in dose, the efficacy of the drug does not always increase and could plateau at a lower dose. In these cases, it is imperative to find a dose that is optimal for both safety and efficacy to maximize the risk-benefit trade-off, i.e., the OBD. BOIN designs provide a complete suite for dose finding in early phase trials, and a consistent way to explore different scenarios in a unified manner with easy access to software [1] to implement most of these designs. Although built upon Bayesian probability models, these BOIN designs are not only operationally simple in general and have good statistical operating characteristics when compared to other dose-finding designs, but also clinically sound especially when communicating with clinical investigators.

BOIN designs are relatively new and have seen rapid development not only in the methods literature but also in the frequency of their implementation in clinical trials. In 2021, the FDA granted the BOIN design the fit-for-purpose designation for dose finding, which has increased its significance and utilization in drug development programs (Drug Development Tools: Fit-for-Purpose Initiative | FDA). Hence, we aim to provide an overview of the current snapshot of this important and rapidly evolving class of cutting-edge dose-finding designs.

**Fig. 1:**
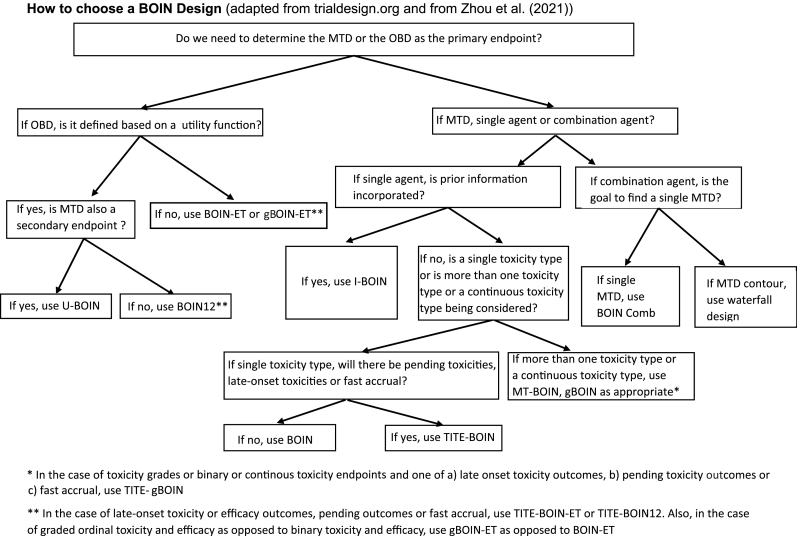
**How to choose a BOIN Design** (adapted from trialdesign.org and from Zhou et al. [1]).

To address the unique practical challenges that arise from the development of precision oncology, several extensions of the original BOIN design have been proposed. In this article, we will provide thumbnail sketches of the original BOIN design and its extensions, discuss their advantages and limitations, list the software used to implement them, provide examples of their use (in the **Appendix**), and detail the situation in which each of the designs in this class is suitable to use (Fig. 1).

In this paper, we do not focus on comparing the BOIN design(s) with other designs, but instead present the different BOIN designs. The following BOIN designs that we consider constitute an up-to-date list. However, this is a rapidly expanding literature and additional designs may be added in the next few years. We further classify the BOIN designs into three categories: the first category only considers toxicity in dose finding to determine the MTD of a monotherapy, the second category uses both toxicity and efficacy to determine the OBD of a monotherapy, and the third category only considers toxicity of a combination therapy to determine either a single MTD or the MTD contour.

In the first category, we review herein: 1) the original BOIN design that considers only the binary endpoint of dose limiting toxicity (DLT); 2) the MT-BOIN design that considers different toxicity types and grades; 3) the gBOIN design that handles continuous and quasi-binary toxicity endpoints and binary toxicity endpoints; 4) the TITE-BOIN design that considers the time to DLT; and 5) the TITE-gBOIN design that considers quasi-Bernoulli toxicity endpoints, but which can be extended to continuous endpoints, and considers the time to the toxicity endpoint.

In the second category, we review herein: 6) the BOIN-ET design that considers DLT and (binary) response; 7) the BOIN12 design which is a 1-stage design incorporating a utility function involving categorical toxicity and efficacy endpoints; 8) the U-BOIN design which is a 2-stage design incorporating a utility function involving categorical toxicity and efficacy endpoints; 9) the gBOIN-ET design that accounts for efficacy and toxicity grades; 10) the TITE-BOIN-ET design that considers the time to both the efficacy and toxicity events; and 11) the TITE-BOIN-12 design that incorporates a utility function with the time to toxicity and efficacy events in dose selection.

Finally, in the third category for combination therapies, we review herein: 12 a) the combination BOIN design that can be used to find the MTD or 12 b) the BOIN waterfall design to find the MTD contour for more than one drug.

## 2 BOIN designs

### 2.1 Toxicity only designs for monotherapy

#### 2.1.1 Standard/basic BOIN

The BOIN design uses the observed DLT rate at each dose for determining the MTD of a new drug.^1^ This phase 1 dose-finding design is implemented in a simple manner, similar to that of the 3 + 3 design, but with better operating characteristics [2]. BOIN also has comparable or better operating characteristics than many model-based designs such as the continual reassessment method (CRM), and other model-assisted designs such as the modified toxicity probability interval (mTPI) and modified toxicity probability interval 2 (mTPI-2) [3] designs (note that the keyboard design [4], an extension of the mTPI design, is equivalent to mTPI-2) [[5], [6], [7], [8], [9]]. This basic BOIN design contains the 3 + 3 design and the accelerated titration design as special cases [10].

##### 2.1.1.1 Design

The BOIN design is an interval-based, model-assisted design. It is constructed using a Bayesian decision-theoretic framework with the aim of minimizing the probability of incorrect dose escalation/de-escalation decisions for each new cohort of patients. The BOIN design requires close collaboration between clinicians and biostatisticians to pre-specify some design parameters, which include the maximum sample size of the trial N, the cohort size (note that Park et al. investigated cohort size deviations in trials using designs such as the BOIN and CRM and showed that some cohort size deviation may be generally acceptable and has little association with the design performance [12]), and the target DLT rate ϕ. Additionally, one also needs to specify a DLT rate ϕ_1_<ϕ and a DLT rate ϕ_2_>ϕ. In general, ϕ_1_ can be treated as the lowest toxicity rate below which a dose is considered sub-therapeutic, and ϕ_2_ can be treated as the highest toxicity rate above which a dose is considered excessively toxic. The recommended default values for ϕ_1_ and ϕ_2_ are ϕ_1_ = 0.6ϕ and ϕ_2_ = 1.4ϕ. Once the design parameters are pre-specified, the optimal lower and upper boundaries, λ_e_ and λ_d_, of BOIN are calculated such that these interval boundaries minimize the incorrect decision of dose escalation and de-escalation. The formulae for λ_e_ and λ_d_ for a non-informative prior, which assumes that each dose has equal prior probability of being at, below or above the MTD, are

λ_e_ = log((1-ϕ_1_)/(1-ϕ))/log((ϕ(1-ϕ_1_))/(ϕ_1_(1-ϕ))), and

λ_d_ = log((1-ϕ)/(1-ϕ_2_))/log((ϕ_2_(1-ϕ))/(ϕ(1-ϕ_2_))).

**Fig. 2:**
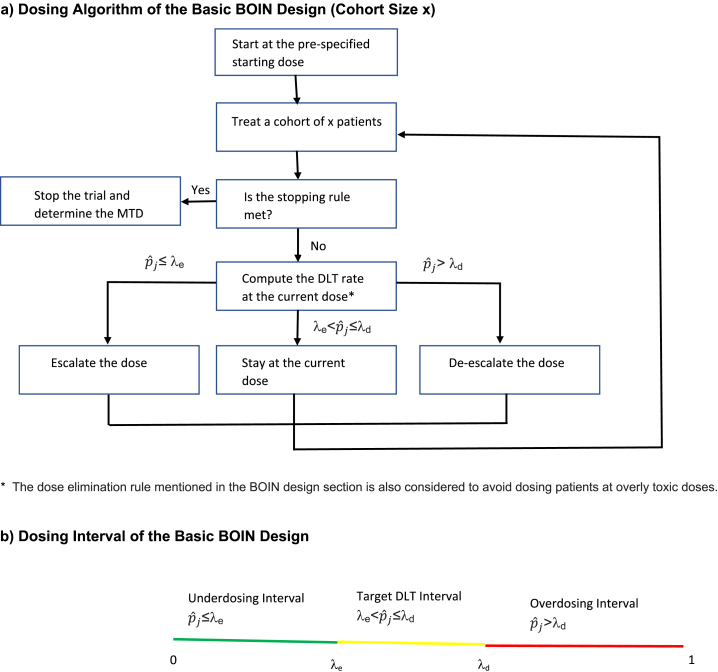
Dosing Algorithm of the Basic BOIN Design (Refer to Section 2.1.1 for details).

Based on the interval boundaries, the specific steps to implement the BOIN design in a phase I dose-finding study are listed below, where p_j_ is the true DLT probability at dose level j, and is estimated by the observed DLT rate ${\hat{p}}_{j}=$ y_j_/n_j_, where y_j_ is the number of patients with DLTs at dose level j and n_j_ is the number of patients treated at dose level j [10]. Fig. 2 a depicts the dosing algorithm of the BOIN design.

1. Treat the first cohort of patients at the lowest dose or the pre-specified starting dose.
2. Calculate the observed DLT rate at the current dose level j ${\hat{p}}_{j}$ (=y_j_/n_j_) (see Fig. 2 b).
  a. If ${\hat{p}}_{j}$ ≤λ_e_, then treat the next cohort of patients at the next higher dose.
  b. If ${\hat{p}}_{j}$ >λ_d_,^2^ then treat the next cohort of patients at the next lower dose.
  c. If λ_e_< ${\hat{p}}_{j}$ ≤λ_d_, then treat the next cohort of patients at the same dose level.
Note that if ${\hat{p}}_{j}$ ≤λ_e_ at the highest dose level j = J, then treat the next cohort of patients at the same dose even if escalation is recommended. If ${\hat{p}}_{j}$ >λ_d_ at j = 1, then treat the next cohort of patients at the same dose even if de-escalation is recommended.
3. The previous step is repeated until the maximum pre-specified sample size N is reached or dose level 1 is found to be too toxic per the dose elimination/overdose control rule described below, in which case no dose level can be selected as the MTD.
4. At the end of the trial, the MTD is determined by first applying isotonic regression to the observed DLT rates to smooth these rates so that they are monotonically non-decreasing [13], and then selecting the dose for which the smoothed DLT rate is closest to the target DLT rate ϕ.

The BOIN design incorporates a dose elimination/overdose control rule [10]. Consider the dose level j. If the posterior probability, which is calculated using the observed DLT rate at dose j, is large, then the dose is deemed to be too toxic and BOIN eliminates this dose and any higher doses from further consideration in the trial.

The mathematical equation for the dose elimination rule to be checked at dose level j is as follows [14]:

If P(p_j_>ϕ|y_j_, n_j_) > 0.95 and n_j_ ≥ 3 (to minimize incorrect elimination of a safe dose level), then dose level j and higher will not be considered further in the trial. P(p_j_>ϕ|y_j_, n_j_) is evaluated based on a beta-binomial model, assuming y_j_| n_j_, p_j_ ∼ Binom(n_j_, p_j_) and p_j_ ∼ Beta(1,1) ∼ Unif(0,1), which is a non-informative, uniform prior. Then the posterior distribution of pj ∼ Beta(y_j_+1, n_j_-y_j_+1) for j = 1, …, J.

##### 2.1.1.2 Software

1) Software to implement the standard BOIN design is available at www.trialdesign.org. As mentioned, the standard BOIN design uses a non-informative approach by assuming that each dose has equal prior probability of being at, below or above the MTD. However, an informative prior based on historical data could also be used so that different amounts of prior information are available for different doses. Software to implement such a design, called the i-BOIN design, is available at www.trialdesign.org.
2) The R package BOIN can be used to implement the standard BOIN design [14].
3) The BOIN design can be implemented using the desktop application available at https://biostatistics.mdanderson.org/SoftwareDownload/SingleSoftware/Index/99.

##### 2.1.1.3 Advantages/limitations

Although built upon Bayesian probability models, the BOIN design is operationally simple, since once λ_e_ and λ_d_ are pre-determined for the given target DLT rate, the dosing decision for each new cohort of patients is based mainly on comparing the observed DLT rate at the current dose with λ_e_ and λ_d_. The BOIN design selects the MTD more accurately and doses a larger percentage of patients at the MTD than the 3 + 3 design does, and it has a lower probability of overdosing patients than some other designs [10].

This basic BOIN design considers only toxicity in its dosing decisions without using efficacy data, which is an important limitation for immuno-oncology drugs where efficacy does not always increase with higher doses. BOIN also does not consider late-onset toxicities or efficacy responses. Finally, BOIN mainly uses the data from the current dose level for dosing decisions and not data collected across all dose levels. However, it is a sequential design where the consecutive dosing decisions indirectly use the information from the adjacent doses, and this results in good statistical operating characteristics. For this same reason, many of the BOIN designs described later also inherit high efficiency and good operating characteristics comparable to model-based designs that use data across all doses.

#### 2.1.2 MT-BOIN

The MT-BOIN (multiple toxicity BOIN) design(s) is an extension of the BOIN design that considers different toxicity types and grades [15].

##### 2.1.2.1 Design

Here, for the dosing algorithm, we focus on one of the cases that MT-BOIN considers, namely that of two non-nested toxicity outcomes^3^ where the two toxicities do not depend on each other. The specific steps of the MT-BOIN design to identify the MTD with two non-nested toxicity outcomes Y_1_ and Y_2_ are as follows [15]:

1. Enroll the first cohort of patients at the lowest dose or starting dose.
2. The number of patients who have experienced toxicity Y_l_ is y_lj_ out of n_j_ treated patients. The toxicity rate at dose level j for toxicity Y_l_ is ${\hat{p}}_{lj}$ = y_lj_/n_j_, l = 1, 2. The dose level assigned to the next cohort of patients is based on comparing ${\hat{p}}_{lj}$ with the pre-specified lower and upper toxicity boundaries λ_le_(n_j_, ϕ_l0_) and λ_ld_(n_j_, ϕ_l0_), l = 1,2. The target toxicity probability of Y_l_ is denoted by ϕ_l0_.
  a) If ${\hat{p}}_{1j}$ ≤ λ_1e_(n_j_, ϕ_10_) and ${\hat{p}}_{2j}$ ≤ λ_2e_(n_j_, ϕ_20_), then the current dose level is safe for both toxicities; escalate the dose and treat the next cohort of patients at dose j+1.
  b) ${\hat{p}}_{1j}$ > λ_1d_(n_j_, ϕ_10_) (or ${\hat{p}}_{2j}$ > λ_2d_(n_j_, ϕ_20_)), then the current dose level is too toxic for outcome Y_1_ (or Y_2_); de-escalate the dose and treat the next cohort of patients at dose j-1.
  c) otherwise stay at this dose and treat the next cohort of patients at dose j.
3. The previous step is repeated until the maximum pre-specified sample size N is reached or the trial is terminated early for safety reasons.
4. At the end of the trial, the MTD is determined by first applying isotonic regression to the estimated toxicity rates ${\hat{p}}_{lj}$ = y_lj_/n_j_, j = 1, …., J to smooth these rates so that they are transformed to monotonically non-decreasing rates ${\tilde{p}}_{lj}$. The MTD dose $\hat{j}$^+^ = min{${\hat{j}}_{l}$*, l = 1, 2}, where ${\hat{j}}_{l}$* = arg min| ${\tilde{p}}_{lj}$ - ϕ_l0_|, jεM (see Ref. [15] for further details), and the set M = {j: n_j_ > 0} contains all the tested dose levels. If there are ties for ${\tilde{p}}_{l{\hat{j}}_{l}*}$,l = 1,2, the dose level tested with the most patients from the tied set is chosen.

For non-nested toxicity outcomes, the optimal interval boundaries of the MT-BOIN design(s) are the same as those of the BOIN design by treating the multiple toxicity outcomes marginally [15]. The MT-BOIN design also deals with nested^3^ toxicity outcomes [15]. The MT-BOIN design has also been extended to drug combinations [15].

##### 2.1.2.2 Software

The boundaries of MT-BOIN are the same as those of the standard BOIN design for non-nested outcomes. Thus, the BOIN software can be used to implement MT-BOIN.

##### 2.1.2.3 Advantages/limitations

MT-BOIN is simple to implement and has comparable operating characteristics to those of model-based designs such as MC-CRM [44] (CRM design that accounts for multiple toxicity constraints) [15]. MT-BOIN is also more robust than model-based designs since it does not rely on a parametric dose-response assumption. In addition to non-nested and nested toxicities, MT-BOIN can be adapted to handle drug combinations [15].

MT-BOIN does not consider late-onset toxicities or efficacy responses.

#### 2.1.3 gBOIN

The gBOIN (generalized BOIN) design [16] is a more general version of BOIN that can handle continuous endpoints such as total toxicity burden^4^ [17], toxicity burden score^5^ [18] or total toxicity profile^6^ [19] and quasi-binary toxicity endpoints such as normalized equivalent toxicity score^7^ [20] and binary endpoints such as DLT. The sample mean of the toxicity endpoint at the current dose j is denoted as ${\hat{\mu }}_{j}=\overset{n_{j}}{\underset{i=1}{\sum }}y_{i}/n_{j}$, where y_1_, …, y_nj_ are the observed toxicity data from n_j_ patients treated at dose d_j_. ${\hat{\mu }}_{j}$ is the observed toxicity rate at dose level j for a binary or quasi-binary endpoint and is the observed sample mean for a continuous endpoint. The dose escalation and de-escalation boundaries λ_e_ and λ_d_ for Bernoulli, quasi-Bernoulli and continuous toxicity endpoints for the parameters ϕ_1_ = 0.6ϕ_0_ and ϕ_2_ = 1.4ϕ_0_ are given in Table 2 of [16]; with ϕ_0_ being the target toxicity value – it is a rate for a binary or quasi-binary endpoint, and a mean for a continuous endpoint.

##### 2.1.3.1 Design

The dosing algorithm of the gBOIN design is as follows and is also given in Fig. 1 of Mu et al. [16]:

1) The first cohort of patients is treated at the lowest dose or at the pre-specified starting dose.
2) Let the current dose level be j.
  a. If ${\hat{\mu }}_{j}$ ≤λ_e_, then treat the next cohort of patients at the next higher dose.
  b. If ${\hat{\mu }}_{j}$ >λ_d_, then treat the next cohort of patients at the next lower dose.
  c. If λ_e_< ${\hat{\mu }}_{j}$ ≤λ_d_, then treat the next cohort of patients at the same dose level.
3) The previous step is repeated until the maximum pre-specified sample size N is reached or dose level 1 is found to be too toxic per the dose elimination rule, in which case no dose level can be selected as the MTD; the same dose elimination rule used in BOIN is also used in gBOIN.
4) At the end of the trial, the MTD is determined by first applying isotonic regression to the observed toxicity values to smooth these values so that they are monotonically non-decreasing [13], and then selecting the dose for which the smoothed toxicity value is closest to the target toxicity value ϕ_0_. If there are ties in the smoothed toxicity values, the highest dose level among the ties for which the smoothed toxicity value is < ϕ_0_ or the lowest dose level among the ties for which the smoothed toxicity value is >ϕ_0_ is chosen.

##### 2.1.3.2 Software

gBOIN can be implemented using the UnifiedDoseFinding R package available at https://cran.r-project.org/web/packages/UnifiedDoseFinding/index.html.

##### 2.1.3.3 Advantages/limitations

The gBOIN design [16] has good statistical operating characteristics compared to existing designs that handle toxicity grades such as the quasi-CRM design [44]. It is simple to implement, since its dosing decisions involve comparing the sample mean of the endpoint with the two pre-specified boundaries of dose escalation and de-escalation, and do not involve any model fitting. It also does not require a lead-in phase and its decision rules can be applied throughout the trial, unlike some model-based designs that need to collect preliminary data through a pre-phase before the model can be reliably estimated [16]. Although the gBOIN design mainly uses data from the current dose level for dosing decisions, it indirectly uses the information from the adjacent doses due to its sequential dose escalation/de-escalation process and its performance is usually as good as or better than the model-based designs that borrow information across doses.

The required elicitation of weights (to determine a toxicity score) and a target involves a time-consuming collaboration between clinicians and biostatisticians. gBOIN does not consider late-onset toxicities or efficacy responses.

#### 2.1.4 TITE-BOIN

In the BOIN design, the next cohort of patients can only be dosed after all the patients in the current cohort have either experienced a DLT or have completed the DLT evaluation period without a DLT. The TITE-BOIN (Time-to-Event-BOIN) design accommodates late onset toxicities and rapid accrual, allowing dosing decisions even with pending DLT data from some of the patients in the current cohort. When there are no pending DLT data, it reverts to the BOIN design [22].

##### 2.1.4.1 Design

The specific steps of the TITE-BOIN design are as follows [22]:

1) Enroll the first cohort of patients at the lowest dose or starting dose.
2) Enroll the next cohort of patients based on the number of patients with DLTs and number of patients with pending DLT data at the current dose and based on a dosing decision table such as Table 1. Here, we chose to show the dosing rules only up to 9 treated patients but the table in Yuan et al. [22], also shows the rules for 12 and 15 patients.

**Table 1:** Dose-escalation and de-escalation rule for TITE-BOIN with a target DLT rate of 0.2 and a cohort size of 3.

Number Treated	Number of DLTs	Number with data pending	STFT		
			Escalate	Stay	De-escalate
3	0	≤1	Y		
3	0	≥2		Suspend Accrual	
3	1	≤2			Y
3	≥2	≤1			Y&Elim
6	0	≤3	Y		
6	0	≥4		Suspend Accrual	
6	1	≤3		Y	
6	1	≥4		Suspend Accrual	
6	2	≤4			Y
6	≥3	≤3			Y&Elim
9	0	≤4	Y		
9	0	≥5		Suspend Accrual	
9	1	≤2	Y		
9	1	3	≥0.77	<0.77	
9	1	4	≥2.15	<2.15	
9	1	≥5		Suspend Accrual	
9	2	0		Y	
9	2	1		>0.52	≤0.52
9	2	2		>1.59	≤1.59
9	2	3		>2.66	≤2.66
9	2	4		>3.73	≤3.73
9	2	≥5		Suspend Accrual	
9	3	≤6			Y
9	≥4	≤5			Y&Elim

NOTE: “Number treated” is the total number of patients treated at the current dose level, “Number of DLTs” is the number of patients who experienced DLT at the current dose level, “Number with data pending” denotes that number of patients whose DLT data are pending at the current dose level, “**STFT**” is the standardized total follow-up time for the patients with data pending, defined as the total follow-up time (TFT) for the patients with data pending divided by the length of the DLT assessment window (example to calculate STFT is shown in the Appendix). “Y" represents “Yes,” and “Y&Elim” represents “Yes and Eliminate.” When a dose is eliminated, all higher doses should also be eliminated [22]. “Suspend accrual” means the following: patient accrual is suspended to await the availability of more data when more than 50% of the patients' DLT outcomes are pending at the current dose [22].
3) Repeat step 2 until the pre-specified maximum sample size is reached. The MTD is determined by first applying isotonic regression to the observed DLT rates to smooth these rates so that they are monotonically non-decreasing, and then selecting the dose for which the smoothed DLT rate is closest to the target DLT rate ϕ.

##### 2.1.4.2 Software

Software to implement the TITE-BOIN design is available at www.trialdesign.org.

##### 2.1.4.3 Advantages/limitations

The TITE-BOIN design is an easy to implement, well-performing dose-finding design that considers late-onset toxicities and rapid accrual, allowing dosing decisions even with pending DLT data from some patients in the current cohort. The design generally shortens the trial duration substantially compared to waiting for the occurrence of pending DLT events. It is more flexible in choosing the target DLT rate and more accurate in MTD selection than the rolling six design [24], a modified 3 + 3 design that allows for accrual of up to 6 patients even if there are pending data in some patients [22]. TITE-BOIN has similar accuracy in MTD selection as the TITE-CRM design, but it has better overdose control and is easier to implement [22].

The TITE-BOIN design uses DLT data only at the current dose for dosing decisions. This is in contrast to the TITE-CRM design, which considers data from all dose levels via a dose-toxicity model. However, simulations show that the effect of using only the current dose data leads to negligible efficiency loss [25, 38]. The TITE-BOIN design assumes that the time to DLT is distributed uniformly over the DLT assessment window, which is similar to what is assumed in the TITE-CRM design. TITE-BOIN is robust to this uniformity assumption, but if reliable prior information is available on the distribution of the time to DLT, then an informative prior can be used to improve design efficiency [22]. TITE-BOIN does not consider efficacy endpoints for its dosing decisions.

#### 2.1.5 TITE-gBOIN

The TITE-gBOIN is a non-parametric, model-assisted design that is an extension of the gBOIN design that accounts for toxicity grades based on both cumulative and pending numeric toxicity scores [26]. Although the reference by Takeda et al. [26] focuses on quasi-Bernoulli toxicity endpoints, the TITE-gBOIN design proposed by the authors can be extended to continuous endpoints. The TITE-gBOIN design minimizes the posterior probability of incorrect dose allocation for patients, while allowing sequential enrollment even with pending toxicity assessment for some patients. If there are no patients with pending toxicity assessments, TITE-gBOIN provides very similar results to those of gBOIN.

##### 2.1.5.1 Design

When there are pending toxicity data, the observed mean toxicity at the current cohort, ${\hat{\mu }}_{j},$ cannot be calculated, as was done for the gBOIN design. However, ${\hat{\mu }}_{j}$ can be replaced by the estimated rate $\;{\tilde{\mu }}_{j}$ based on the likelihood with pending quasi-Bernoulli toxicity data - ${\tilde{\mu }}_{j}$ is the maximum likelihood estimate of $\mu_{j}.$ Once ${\tilde{\mu }}_{j}$ is obtained, the dosing algorithm of the gBOIN design can be used with ${\tilde{\mu }}_{j}$ taking the place of ${\hat{\mu }}_{j}$ in the gBOIN algorithm. If patient accrual is much faster than outcome evaluation, a rule that suspends dose allocation until there is adequate information may need to be employed. For example, accrual can be suspended to await the availability of more data when more than 50% of the patients’ toxicity outcomes are pending at the current dose.

In this design, the estimated quasi-Bernoulli toxicity probability ${\tilde{\mu }}_{j}$ is updated until all the patients have completed their toxicity assessment. At the end of the trial, the MTD is determined by first applying isotonic regression to the estimated quasi-Bernoulli toxicity probabilities to smooth these values so that they are monotonically non-decreasing, and then selecting the dose for which the smoothed quasi-Bernoulli toxicity probability is closest to the target quasi-Bernoulli toxicity probability ϕ_0_.

##### 2.1.5.2 Software

Software to implement the TITE-gBOIN design is available at https://github.com/qingxiaa/titegboin.

##### 2.1.5.3 Advantages/limitations

The TITE-gBOIN design is a robust design that is simple to implement. Simulations in various realistic scenarios show that the TITE-gBOIN design is comparable in performance to the gBOIN design and it has a higher probability of selecting the MTD correctly and allocating more patients to the MTD than other available approaches [26]. It reduces trial duration compared to designs that do not allow sequential dose allocation.

The design performance may depend on the appropriate specification of the quasi-Bernoulli endpoint such as the normalized equivalent toxicity score (ETS) [26]. A time-consuming collaboration between clinicians and biostatisticians is required to accurately derive the quasi-Bernoulli endpoint utilizing the weight for each toxicity grade and the drug-specific toxicity profiles [26]. The clinician needs to consider the clinical meaning while assigning the quasi-Bernoulli endpoint to a given grade of toxicity, while the biostatistician needs to evaluate the robustness of the quasi-Bernoulli endpoint being considered via simulations [26]. TITE-gBOIN does not consider efficacy responses.

### 2.2 Toxicity and efficacy designs for monotherapy

#### 2.2.1 BOIN-ET

For some targeted therapies, such as biological agents and immunotherapies, the efficacy may not necessarily increase with an increase in dose. Hence, it is necessary to determine the OBD by considering both toxicity and efficacy. The BOIN-ET (BOIN Efficacy Toxicity) design, a phase I/II model-assisted design, is an extension of the BOIN design that utilizes both binary efficacy and toxicity outcomes in determining the OBD [27]. The STEIN design is an extension of the BOIN design that considers drug efficacy in addition to toxicity and is based on the BOIN design boundaries [28]. While similar to BOIN-ET in its dosing algorithm, STEIN independently identifies the optimal decision boundaries for toxicity and efficacy, whereas BOIN-ET jointly optimizes these parameters.

##### 2.2.1.1 Design

Assuming the current cohort of patients is treated at dose level j, the BOIN-ET design is implemented as described below (the algorithm is also shown in Table 2) [27]. For toxicity, ${\hat{p}}_{j}$ is the observed DLT rate at dose level j. ϕ is the pre-specified target DLT rate with the lower and upper boundary of the target DLT interval being λ_e_ and λ_d_, respectively. For efficacy, q_1_, …..,q_j_ are the true efficacy probabilities at each dose level j, estimated by ${\hat{q}}_{j}$ = x_j_/n_j_, the observed response rate at dose level j, where x_j_ is the number of patients with responses observed and n_j_ is the number of patients treated at dose level j. δ is the pre-specified target efficacy probability and η_1_ is the efficacy cut-off satisfying 0≤ η_1_<δ < 1 and is computed numerically.

1) If ${\hat{p}}_{j}$ ≤ λ_e_ and ${\hat{q}}_{j}$ ≤ η_1_, dose the next cohort of patients at dose level j+1.
2) If ${\hat{p}}_{j}$ ≤ λ_d_ and ${\hat{q}}_{j}$ > η_1_, dose the next cohort of patients at the same dose level j.
3) If ${\hat{p}}_{j}$ > λ_d_, dose the next cohort of patients at dose level j-1.
4) If λ_e_ < ${\hat{p}}_{j}$ ≤ λ_d_ and ${\hat{q}}_{j}$ ≤ η_1_, due to the possibility of a non-monotonic dose-efficacy relationship, we define a set of admissible doses A_j_=(j-1, j, j+1).
  o We select a dose from the admissible dose levels j − 1, j, j + 1 for the next cohort as follows:
    a) If dose level j + 1 has not been used earlier, escalate the dose to level j + 1.
    b) If the above point does not apply, choose the dose that has the maximum probability of efficacy according to ${\hat{q}}_{j-1}$, ${\hat{q}}_{j}$, and ${\hat{q}}_{j+1}$.
    c) If neither point above applies because the doses share the same estimated maximum probability of efficacy, randomly choose 1 dose among the doses that share the maximum probability of efficacy.

**Table 2:** Dosing Decision Table for the BOIN-ET Design.

	0≤ ${\hat{p}}_{j}$ ≤ λ_e_	λ_e_ < ${\hat{p}}_{j}$ ≤ λ_d_	λ_d_ < ${\hat{p}}_{j}$ ≤ 1
$\eta_{1}<{\hat{q}}_{j}\leq 1$	Stay	Stay	De-escalate
$0\leq {\hat{q}}_{j}$ ≤ η_1_	Escalate	Escalate/Stay/De-escalate	De-escalate

The optimal values of the three parameters λ_e_, λ_d_ and η_1_ to be used in the dosing decisions cannot be derived analytically but can be computed numerically. Once these are estimated, the dosing rules of the trial can be pre-specified, as in Table 3, which uses values of λ_e_, λ_d_ and η_1_ estimated to be 0.14, 0.35 and 0.48 [27].

**Table 3:** Example Dose Finding Table for the BOIN-ET Design.

		Cumulative Number of Responses						
		0	1	2	3	4	5	6
Cumulative Number of DLTs	0	E	E	E	S	S	S	S
	1	E/S/D	E/S/D	E/S/D	S	S	S	S
	2	E/S/D	E/S/D	E/S/D	S	S	S	S
	3	D	D	D	D	D	D	D
	4	D	D	D	D	D	D	D
	5	D	D	D	D	D	D	D
	6	D	D	D	D	D	D	D

D = De-Escalate, E = Escalate, S=Stay. The target toxicity probability and efficacy probability assumed are ϕ = 0.3 and δ = 0.6 respectively. The assumed design parameters of ϕ_1_ = 0.1ϕ, ϕ_2_ = 1.4ϕ and δ_1_ = 0.6δ lead to optimal values of λ_e_, λ_d_ and η_1_ of 0.14, 0.35 and 0.48.

At the end of the trial, isotonic regression is applied to smooth the observed toxicity rates so that they are monotonically non-decreasing. The MTD is selected as the dose for which the isotonic DLT rate is closest to the target DLT rate ϕ. For efficacy, fractional polynomials with 2 degrees of freedom, to allow non-monotonic dose-response relationships, are used to fit the data. A subset of J dose levels B = {j: j ≤ m} satisfying the tolerability, where the mth dose is the estimated MTD, is defined. Among set B, the OBD is the dose j* that maximizes the efficacy probability.

##### 2.2.1.2 Software

Takeda et al. provide SAS code upon request to implement the BOIN-ET design. The following reference provides example dosing decision tables for cohort sizes of 3 and 6 to implement the BOIN-ET design [27].

##### 2.2.1.3 Advantages/limitations

The statistical operating characteristics of the BOIN-ET design have been compared to those of other designs [27], such as the design proposed by Thall and Cook (TC method [30]), and the design proposed by Sato, Hirakawa, and Hamada (SHH method, [31]). In general, the BOIN-ET design selects the OBD more accurately and puts a higher average number of patients at the OBD than the model-based, Bayesian adaptive TC and SHH designs [27]. BOIN-ET is simpler and easier to implement than these designs, is safer, and provides much better overdosing control [27].

However, when the efficacy rate is sufficient at lower doses, the BOIN-ET design tends to allocate more patients to doses higher than the OBD, because of its forced escalation when ${\hat{p}}_{j}$ ≤ λ_e_ and ${\hat{q}}_{j}$ ≤ η_1_. Thus, if the true OBD is at lower doses, BOIN12 (described below), which is more conservative in dose escalation than BOIN-ET, will select the OBD more accurately (and vice-versa when the true OBD is at higher doses). Further, BOIN-ET may not be suitable for solid tumors, where the RECIST criteria are used for the efficacy assessment [32]. In this case, it may cause delays in implementing the dosing decisions because the RECIST efficacy evaluation usually occurs later than the toxicity evaluation. Hence, this design may be most applicable to oncology trials where the response assessment period and the DLT assessment period are on similar time scales, assuming that the short-term binary response used in the trial decision making can predict long-term responses such as overall survival.

#### 2.2.2 BOIN12

BOIN12 is a flexible phase I/II model-assisted design to determine the OBD by optimizing the risk-benefit tradeoff. Each cohort of patients is allocated to the dose that maximizes the toxicity-efficacy trade-off [21]. BOIN12 uses categorical toxicity and efficacy data in a single stage to determine the OBD. A posterior interval estimator is employed to determine the next dose assignment.

##### 2.2.2.1 Design

The specific steps of the BOIN12 dosing algorithm for a binary efficacy and a binary toxicity endpoint are described below; the flow diagram for the BOIN12 dosing algorithm is also given in Fig. 1 of Lin et al. [21].

1. Treat the first cohort of patients at the lowest dose or pre-specified starting dose.
2. p_1_, …..,p_j_ are the true DLT probabilities and q_1_, …..,q_j_ are the true efficacy probabilities at each dose level j. The observed DLT rate at dose level j is ${\hat{p}}_{j}$, and it is equal to the number of patients who experience a DLT at dose level j divided by n_j_, the number of patients treated at dose level j. The observed response rate at dose level j is ${\hat{q}}_{j}$, and it is calculated similarly. The lower boundary of the pre-calculated target DLT interval is λ_e_ and the upper boundary is λ_d_, based on the BOIN design and ϕ_T_. ϕ_T_ and ϕ_E_ are the pre-specified toxicity upper limit and efficacy lower limit, respectively. Generally, ϕ_T_ should be set at a value slightly higher than the target toxicity rate used in conventional toxicity-based phase I trials, and ϕ_E_ can take the value of the alternate response rate specified for a standard phase II trial.
  a) If ${\hat{p}}_{j}$ >λ_d_ then treat the next cohort of patients at the next lower dose j-1.
  b) If λ_e_< ${\hat{p}}_{j}$ and n_j_ ≥ 6, then determine the desirability of doses j and j-1 using the pre-specified RDS (rank-based desirability score) table (e.g. Table 3 in Ref. [21]; a part of which is reproduced here (Table 4)). The RDS is based on a utility score elicited from clinicians. A larger value of RDS implies a higher utility. The mean utility at dose j is given by u(j) = Ψ_1_u_1_+ Ψ_2_u_2_+ Ψ_3_u_3_+ Ψ_4_u_4_, where u_1_ (=100), u_2_ (= an elicited score between 0 and 100), u_3_ (=an elicited score between 0 and 100^8^) and u_4_ (=0) denote the utility scores ascribed to the outcomes of 1) efficacy and no toxicity, 2) no toxicity and no efficacy, 3) both toxicity and efficacy and 4) toxicity and no efficacy respectively, and Ψ_1_, Ψ_2_, Ψ_3_, Ψ_4_ denote the probabilities of observing these outcomes at dose j - note: Ψ_1j_ +Ψ_3j_ = q_j_ and Ψ_3j_ +Ψ_4j_ = p_j_. The utility function used is flexible; details on how RDS is calculated based on the utility is provided in Lin et al. [21]. Treat the next cohort of patients at the dose that has the highest desirability.

**Table 4:** Decision Table (RDS Table) for BOIN12 assuming the upper toxicity limit ϕ_T_ to be 0.35 and the lower efficacy limit ϕ_E_ to be 0.25 and the utility specification given in Table 1 of Lin et al. [21].

No of Patients	No of Toxicities	No of Efficacies	Desirability Score
0	0	0	60
3	0	0	35
3	0	1	55
3	0	2	76
3	0	3	91
3	1	0	24
3	1	1	44
3	1	2	63
3	1	3	80
3	2	0	13
3	2	1	31
3	2	2	48
3	2	3	69
3	3	Any	ED
6	0	0	22
6	0	1	38
6	0	2	51
6	0	3	67
6	0	4	81
6	0	5	93
6	0	6	100
6	1	0	15
6	1	1	27
6	1	2	42
6	1	3	56
6	1	4	72
6	1	5	87
6	1	6	96
6	2	0	8
6	2	1	19
6	2	2	34
6	2	3	47
6	2	4	64
6	2	5	77
6	2	6	90
6	3	0	4
6	3	1	12
6	3	2	22
6	3	3	38
6	3	4	51
6	3	5	67
6	3	6	81
6	4	0	1
6	4	1	6
6	4	2	15
6	4	3	27
6	4	4	42
6	4	5	56
6	4	6	72
6	≥5	Any	ED

ED means the dose should be eliminated because it does not satisfy the safety and efficacy admissible criteria (i.e., not admissible because of high toxicity or low efficacy).
  c) If ${\hat{p}}_{j}$ ≤λ_e_ or if λ_e_< ${\hat{p}}_{j}$ ≤λ_d_ and n_j_ < 6, then determine the desirability of dose levels j, j-1 and j+1 using the pre-specified RDS table. Treat the next cohort of patients at the dose that has the highest desirability.
3. The previous step is repeated until the maximum pre-specified sample size N is reached.
4. The OBD is then selected based on the following 2-step procedure:
  a) The MTD is determined by first applying isotonic regression to smooth the observed DLT rates so that they are monotonically non-decreasing, and then selecting the dose for which the smoothed DLT rate is closest to the pre-specified toxicity upper limit ϕ_T_.
  b) The final OBD is the dose level that has the highest estimated utility value of those doses not higher than the MTD.

##### 2.2.2.2 Software

Software to implement the BOIN12 design is available at www.trialdesign.org.

##### 2.2.2.3 Advantages/limitations

BOIN12 is based on a utility trade-off function and is more general, while BOIN-ET is based on marginal toxicity and marginal efficacy rates and does not incorporate a toxicity-efficacy utility trade-off function in dose finding. The BOIN12 design is simple to implement, and it selects the OBD more accurately and doses more patients at the OBD compared to dose-finding designs such as the TC method [30], TEPI (Toxicity Efficacy Probability Interval) [33] and 3 + 3 CE (cohort expansion) (i.e. a 3 + 3 design followed by a CE at the identified MTD, with Simon's two-stage design used to monitor efficacy in CE) designs. The dosing decision table for the BOIN12 design can be used to make dosing decisions and allocate patients to a dose without any complex calculations [21].

In some immunotherapy trials, late onset toxicities and responses may be observed. This will preclude using the BOIN12 design, since it assumes that the toxicity and efficacy outcomes are available by the time of the dose assignment of the next cohort.

#### 2.2.3 U-BOIN

The utility BOIN (U-BOIN) design can be used to determine the OBD of the drug considering both efficacy and toxicity data. It is a two-stage utility-based, seamless Bayesian phase I/II model-assisted design [34]. Only toxicity data are used in the first stage, and both toxicity and efficacy data are used in the second stage to determine the OBD. U-BOIN can handle categorical efficacy and toxicity endpoints, but in the below section, we assume that the efficacy and toxicity endpoints are binary. A posterior point estimator is employed in the dosing decisions.

##### 2.2.3.1 Design

The specific steps of the U-BOIN design are described below and are also depicted in Fig. 1 of Zhou et al. [34].

The U-BOIN design comprises two seamless stages.

Stage 1 is the same as the BOIN design and its aim is to identify a set of admissible doses for Stage 2. In Stage 1, the dose finding is based only on DLT data although efficacy data are also collected. In Stage 1, the trial proceeds exactly as the BOIN design. Once the number of patients treated at one of the doses reaches the pre-specified maximum sample size s1, the trial proceeds to Stage 2.

In Stage 2, the efficacy and toxicity data from both stages are used to determine the OBD for efficacy and toxicity. Note that ${\hat{\pi }}_{T,\;j}$ = m_j_/n_j_ is the observed DLT rate at dose level j, where m_j_ is the number of patients who experienced a DLT at dose level j and n_j_ is the number of patients treated at dose level j. λ_e_ and λ_d_ are the pre-determined optimal escalation boundary and de-escalation boundary for the BOIN design based on the considered target DLT rate.

In Stage 2, the trial proceeds as follows:

1) Let j* be the highest dose that has been tried. If ${\hat{\pi }}_{T,\;j^{*}}$ ≤ λ_e_ and j* is not the highest dose level in the trial, then assign the next cohort of patients to j*+1. If not, proceed to step 2.
2) Determine the admissible set of doses based on the data D observed thus far in stages 1 and 2. Assign the next cohort of patients to the dose that has the largest posterior mean utility value among the admissible doses. If there is no admissible dose, terminate the trial – there is no OBD.

A dose is inadmissible if either of the following criteria is satisfied:

$$\text{Pr}\left(\pi_{\text{T,j}}>{\bar{\pi }}_{T}|\text{D}\right)\text{>}{\text{C}}_{\text{T}}\;\text{or}\;\text{Pr}\left(\pi_{\text{E,j}}\text{<}{\underline{\pi }}_{E}|\text{D}\right)\text{>}{\text{C}}_{\text{E}}\text{,}$$

where π_T,j_ is the marginal probability of toxicity at dose level j, ${\bar{\pi }}_{T}$ is the maximum tolerable DLT rate, π_E,j_ is the marginal probability of efficacy at dose level j, and ${\underline{\pi }}_{E}$ is the lowest acceptable response rate. C_E_ and C_T_ are probability cutoffs. In general, C_T_ = 0.95 and C_E_ = 0.9.

3) Repeat steps 1 and 2 until the maximum pre-specified sample size N is reached or the number of patients treated at one of the doses reaches the pre-specified maximum sample size s2 (s2>s1).
4) The OBD is the dose among the admissible doses with the largest posterior mean utility value.

##### 2.2.3.2 Utility function

The utility function is used for dosing decisions in Stage 2 and for determining the OBD.

The true mean utility for dose level j is given as follows:

$${\text{U}}_{\text{j}}=\overset{K}{\underset{k=1}{\sum }}\varphi_{k}\pi_{jk}\text{.}$$

To define $\pi_{jk}$, consider the following outcomes:

Y = 1 = (1, 0) = patient experiences DLT and no response and $\varphi_{1}$ is the weight given in consultation with clinicians to this outcome of toxicity and response and is usually 0.
Y = 2 = (0, 0) = patient experiences no DLT and no response and $\varphi_{2}$ is the weight given in consultation with clinicians to this outcome of toxicity and response.
Y = 3 = (1, 1) = patient experiences DLT and a response and $\varphi_{3}$ is the weight given in consultation with clinicians to this outcome of toxicity and response.
Y = 4 = (0, 1) = patient experiences no DLT and a response and $\varphi_{4}$ is the weight given in consultation with clinicians to this outcome of toxicity and response and is usually 100.

Define

$\pi_{jk}=\mathrm{Pr}\left(Y=k|d=j\right),$ k = 1, …, K (here K = 4) and d is the dose level and goes from j = 1, …, J, and $\overset{K}{\underset{k=1}{\sum }}\pi_{jk}=1$.

We assume that Y follows a Dirichlet-multinomial model. At an interim decision time, we assume that n_j_ patients have been treated at dose d = j, among which n_jk_ patients have outcome Y = k, where n_j_ = $\overset{K}{\underset{k=1}{\sum }}n_{jk}$. Given the observed interim data Dj=($n_{j1},\;n_{j2}$, …, $n_{jK}$), the posterior distribution of $\pi_{j}$ = ($\pi_{j1},\;\pi_{j2}$, …, $\pi_{jK}$) is $\pi_{j}|D_{j}$ and follows a Dirichlet distribution.

The true mean utility U_j_ depends on π_j,k_, which is unknown. The mean utility ${\hat{U}}_{j}$ is estimated based on the observed interim data D = {D_j_} as follows:

$${\hat{U}}_{j}=\overset{K}{\underset{k=1}{\sum }}\varphi_{k}E\left(\pi_{jk}|D\right)\text{.}$$

The OBD is the admissible dose that has the highest utility value.

OBD = argmax_jεA_U_j_, where A is the admissible set.

##### 2.2.3.3 Software

Software to implement the U-BOIN design is available at www.trialdesign.org.

##### 2.2.3.4 Advantages/limitations

The U-BOIN design is simple to implement and is well-performing. It can be implemented in a trial using pre-determined decision tables and does not require complex model fitting and estimation [35]. Compared to a model-based design such as the TC method, U-BOIN identifies the OBD more accurately and is more robust [34]. In addition, due to the incorporation of the first stage, U-BOIN estimates the MTD accurately.

The U-BOIN design models efficacy and toxicity at each dose independently while model-based designs, such as the TC method, model efficacy and toxicity across all doses via a parametric model for dose-efficacy and dose-toxicity curves. Thus, there may be a potential efficiency loss for U-BOIN, although this loss is believed to be minimal or negligible [34]. U-BOIN is comparable to BOIN12 in accuracy of OBD selection, but it requires a relatively large sample size to guarantee a desirable performance due to the use of two stages. The sample size required by U-BOIN is usually greater than the sample size used in MTD-finding trials. In stage 2, U-BOIN assumes that both the toxicity and efficacy outcomes are available by the time of the dose assignment of the next cohort.

#### 2.2.4 gBOIN-ET

The gBOIN-ET design is a phase I/II model-assisted, non-parametric design that is an extension of the BOIN-ET design and that accounts for ordinal graded efficacy and toxicity [36]. Although the reference by Takeda et al. focuses on quasi-Bernoulli toxicity and efficacy endpoints, gBOIN-ET can be extended to continuous endpoints. gBOIN-ET aims to minimizes the posterior probability of incorrect dose allocation for patients regarding efficacy and toxicity [36]. While BOIN12 and gBOIN-ET can handle categorical toxicity and efficacy endpoints, BOIN-12 uses an efficacy-toxicity utility function to determine the OBD, while gBOIN-ET does not.

##### 2.2.4.1 Design

The quasi maximum likelihood estimators for the quasi-Bernoulli endpoints are equal to the observed average quasi-Bernoulli endpoints at each dose level. Thus, the observed toxicity probability and the observed efficacy probability in BOIN-ET can be replaced by the observed quasi-Bernoulli toxicity probability and the observed quasi-Bernoulli efficacy probability respectively, using the quasi-Bernoulli likelihood [36]. The dosing decision rules of BOIN-ET can then be implemented using the observed quasi-Bernoulli toxicity probability and the observed quasi-Bernoulli efficacy probability. At the end of the trial, isotonic regression is applied for toxicity and the regression model with the fractional polynomial is applied for efficacy, in order to determine the OBD [36]. Further details of the dosing algorithm, calculation of the Bayesian optimal boundaries, early termination criteria and OBD selection are given in Takeda et al. [36].

##### 2.2.4.2 Software

SAS code is available to implement the gBOIN-ET design.

##### 2.2.4.3 Advantages/limitations

The gBOIN-ET design is simple and easy to implement in oncology trials than model-based approaches. Simulations used to investigate the operating characteristics of gBOIN-ET show that it has a higher performance than the other designs investigated (BOIN12, gBOIN, BOIN-ET) in terms of the correct OBD selection, the average number of patients allocated to the OBDs, not selecting overdoses as the OBDs and not assigning patients to overdoses [36].

The design performance may depend on the appropriate specification of the quasi-Bernoulli endpoints [36]. A time-consuming collaboration between clinicians and biostatisticians is required to accurately derive the quasi-Bernoulli endpoints utilizing the weight for each toxicity grade and efficacy grade [36]. gBOIN-ET may select lower doses as OBDs if the low quasi-Bernoulli efficacy probability is mis-specified as the target quasi-Bernoulli efficacy probability [36]. Hence clinicians and biostatisticians need to consider target quasi-Bernoulli efficacy and toxicity probabilities that are realistic for the study drug. gBOIN-ET does not consider the accrual rate, the outcome evaluation period and the late-onset outcomes [36]. It may also be valuable to incorporate historical and personalized information into the gBOIN-ET design to improve the efficiency of phase I/II dose-finding trials [36].

#### 2.2.5 TITE BOIN-ET

The TITE-BOIN-ET is a model-assisted design that considers cumulative and pending toxicity and efficacy data. The TITE-BOIN-ET (Time-to-Event-BOIN Efficacy Toxicity) design is an extension of the BOIN-ET design that has been proposed to consider the following factors in dose finding: 1) fast accrual rates, 2) the difference in evaluation periods for toxicity and efficacy, and 3) the late onset outcomes [37].

##### 2.2.5.1 Design

When there are pending toxicity and efficacy data, the observed DLT and response rates at the current cohort cannot be calculated, as was done for the BOIN-ET design. However, the observed DLT and response rates can be replaced by the estimated DLT and response rates based on the likelihood with pending toxicity and efficacy data. As a result, a dosing decision table similar to that used for the BOIN-ET design can be employed. The use of such a table does not require any model fitting and accounts for the pending efficacy and toxicity data. Table 5 is for a cohort size of 3, and a target toxicity probability and target efficacy probability of ϕ = 0.3 and δ = 0.6 respectively [37]. The assumed design parameters of ϕ_1_ = 0.1ϕ, ϕ_2_ = 1.4ϕ and δ_1_ = 0.6δ lead to optimal values of λ_e_, λ_d_ and η_1_ of 0.13, 0.35 and 0.48, respectively.

**Table 5:** Dosing Decision Rules for the TITE-BOIN-ET Design.

	Cumulative number of responses				
Cumulative number of toxicities		0	1	2	3
	0	E	E if ESS_Ej_ >2.07 S if ESS_E j_ ≤ 2.07	S	S
	1	E/S/D if ESS_Tj_ ≥2.87^a^ D if ESS_Tj_<2.87	E/S/D if ESS_Ej_ >2.07 and ESS_Tj_ ≥2.87^a^ S if ESS_Ej_ ≤2.07 and ESS_Tj_ ≥2.87, D if ESS_Tj_<2.87	S if ESS_Tj_ ≥2.87 D if ESS_T j_ < 2.87	S if ESS_Tj_ ≥2.87 D if ESS_T j_ < 2.87
	2	D	D	D	D
	3	D	D	D	D

E = Escalate, D = De-escalate and S=Stay.

a The rules used in the E/S/D cases in TITE-BOIN-ET to decide whether to escalate, de-escalate or stay at the same dose are the same as those used in BOIN-ET for the E/S/D case to decide whether to escalate, de-escalate or stay at the same dose (see BOIN-ET section).

The effective sample size for efficacy at dose j is

$$ESS_{Ej}=Number\;of\;non-pending\;patients\;for\;efficacy\;at\;dose\;level\;j+\frac{\text{Total Follow}-\text{up time for pending patients for efficacy at dose level j}}{\text{Length of assessment window for efficacy}}.$$

The effective sample size of toxicity at dose j is

$$ESS_{Tj}=Number\;of\;non-pending\;patients\;for\;toxicity\;at\;dose\;level\;j+\;\frac{\text{Total Follow}-\text{up time for pending patients for toxicity at dose level j}}{\text{Length of assessment window for toxicity}}.$$

Table 5 can be used directly to determine to which dose level the next cohort of patients should be assigned, once the number of patients with DLTs and responses at dose level j are known and the effective sample sizes for efficacy and toxicity at dose j are calculated.

The estimated toxicity rate and the estimated efficacy rate are updated until the patients complete the toxicity and efficacy assessment periods even without new enrollment [37]. At the end of the trial, isotonic regression is applied so that the estimated toxicity probabilities are monotonically non-decreasing. The MTD is selected as the dose whose isotonic regression estimator is closest to the target DLT rate ϕ. For efficacy, fractional polynomials with 2 degrees of freedom to allow non-monotonic dose-response relationships are used to fit the data. A subset of J dose levels, B = {j: j ≤ m}, satisfying the tolerability where the mth dose is the estimated MTD, is defined. Among the set B, the OBD is the dose j* that maximizes the efficacy probability (the efficacy probabilities are those estimated by the fractional polynomial).

##### 2.2.5.2 Software

Takeda et al. provide SAS code upon request to implement the TITE-BOIN-ET design. The following reference provides an example dosing decision table for cohort size 3 to implement the TITE-BOIN-ET design [37].

##### 2.2.5.3 Advantages/limitations

The TITE-BOIN-ET design is robust, much simpler, and easier to implement than model-based approaches. A simulation study across a range of realistic settings shows that the TITE-BOIN-ET design selects the OBD more accurately and puts a higher average number of patients at the OBD than model-based approaches such as the design by Thall and Cook and the design by Jin et al. [37,43]. The trial duration is also significantly shortened when using the TITE-BOIN-ET design compared to using designs without sequential enrollment [37].

However, when the efficacy response rate is sufficient at lower doses, the TITE-BOIN-ET design tends to allocate more patients to doses higher than the OBD, similar to BOIN-ET. If patient accrual is faster than the outcome evaluation, then the available information may still not be sufficient even if the pending data are considered. In such a case, suspension rules as in Lin and Yuan [25] (e.g. dose escalation is not allowed if fewer than 2 patients at any dose level have completed their assessment) or as in Yuan et al. [22] (see footnote of Table 1 for the suspension rule used in Ref. [22]) may need to be considered to delay the dosing decisions until adequate information is available [37].

#### 2.2.6 TITE-BOIN12

When there are pending outcomes for toxicity or response, the BOIN12 design is not an option as it cannot calculate the DLT probability and dose desirability. The TITE-BOIN12 design is a utility-based phase I/II model-assisted design that deals with late-onset toxicities and responses, allowing the study to proceed with dosing the next cohort of patients even in the presence of pending outcomes for toxicity or response for some patients [39]. TITE-BOIN12 reduces to the BOIN12 design when there are no pending outcomes for toxicity or response.

##### 2.2.6.1 Design

In this design, either Bayesian data augmentation (BDA) or the approximated likelihood method can be used in the dose-finding decisions when there are pending outcomes for some patients for toxicity or response. Using BDA or the approximated likelihood method, the same dose-finding algorithm can be used for TITE-BOIN12 as with BOIN12, with a few changes. At each interim analysis, the TITE-BOIN12 design updates the estimate for admissibility criteria, the marginal DLT probability and the dose desirability for all doses. TITE-BOIN12 also has the additional following accrual suspension rule: if more than 50% of the patients have pending DLT or efficacy outcomes at the current dose, the trial needs to be suspended until more data become available. Further details of the dosing algorithm are given in Zhou et al. [39].

##### 2.2.6.2 Software

Software to implement the TITE-BOIN12 design is available at www.trialdesign.org.

##### 2.2.6.3 Advantages/limitations

TITE-BOIN12 is a well-performing design that allows continuous accrual while still ensuring patient safety and accuracy of OBD identification. In most cases, this design has better over-dose control and higher accuracy of OBD identification, than model-based designs such as the TC method [39]. TITE-BOIN12 accommodates different shapes of dose-efficacy curves compared to the TC method. It shortens the trial duration and incorporates risk-benefit trade-off with input on the utility values from clinicians [39].

TITE-BOIN12 assumes that the time to DLT and efficacy are distributed uniformly over the assessment window, while calculating the STFT, which is similar to what is assumed in the TITE-BOIN and TITE-CRM designs [39]. This design is robust to this assumption, similar to what is demonstrated for TITE-CRM [40] and for TITE-BOIN [22]. However, if reliable prior information is available on the distribution of the time to DLT or response, an informative prior can be used for either or both to improve design efficiency. No decision table can be generated for TITE-BOIN12 prior to the trial conduct due to the large number of possible values for the STFT. However, dose desirability can be easily calculated based on interim data using existing software to determine the dose assignment for the next cohort of patients.

## 3 Combination designs

Drug combination provides an appealing way to obtain synergistic treatment effects and overcome resistance of monotherapy in oncology [14]. Trials to identify the MTD for combined therapies are more complicated than monotherapy trials, due to the higher dimensionality of the dose space and the partially-known toxicity order between the combined doses. Combination BOIN design(s) for phase I trials allows dose finding in 2 dimensions [29]. Assume that the trial tests J doses of drug A with A_1_ < A_2_ < ….< A_J_ (A_1_ is the lowest dose and A_J_ is the highest) and K doses of drug B with B_1_ < B_2_ < ….< B_K_ (B_1_ is the lowest dose and B_K_ is the highest), and p_jk_ is the true DLT rate of the combination of A_j_ and B_k_ denoted as A_j_B_k_. The toxicities probabilities are only partially ordered because dose combination A_j’_B_k’_ will be more toxic than dose combination A_j_B_k_ if j’>j and k’>k but this may or may not be true if j’>j but k’<k [14]. At the end of the trial, either a single MTD or multiple MTDs (MTD contour) can be selected, depending on the trial and its application [23].

### 3.1 Design

#### 3.1.1 BOIN comb design to find single MTD

The pre-specified target DLT rate is ϕ, the lower boundary of the pre-specified (pre-calculated) target DLT interval is λ_e_, and the upper boundary of the target DLT interval is λ_d_. The pre-specified maximum sample size of the trial is N. A_j_B_k_ is the current dose level. The observed DLT rate at A_j_B_k_ is ${\hat{p}}_{jk}$ = y_jk_/n_jk_, where y_jk_ is the number of patients with DLTs observed at dose A_j_B_k_ and n_jk_ is the number of patients treated at dose A_j_B_k_. Define an admissible dose escalation set as A*E* = {*A*_*j*+1_*B*_*k*_*, A*_*j*_*B*_*k*+1_} and an admissible dose de-escalation set as A*D* = {*A*_*j*−1_*B*_*k*_*, A*_*j*_*B*_*k*−1_}. Also for dose-escalation, de-escalation decisions, consider P(p_jk_ ε (λ_e_, λ_d_)|D_jk_), which measures how likely it is that a dose combination is located within the acceptable toxicity interval (λ_e_, λ_d_), where D_jk_=(y_jk_, n_jk_).

The specific steps of the BOIN combination design for finding a single MTD [29] are as follows:

1. Treat the first cohort of patients at the lowest dose combination or a pre-specified dose combination.
2. If the current dose level is A_j_B_k_ and the observed DLT rate is ${\hat{p}}_{jk}$, then assign the next cohort of patients as follows:

If ${\hat{p}}_{jk}$ ≤ λ_e_, then escalate and treat the next cohort of patients at that dose in AE that has the largest value of P(p_j'k’_ ε (λ_e_, λ_d_)|D_jk_).

If ${\hat{p}}_{jk}$ >λ_d_, then de-escalate and treat the next cohort of patients at that dose in AD that has the largest value of P(p_j'k’_ ε (λ_e_, λ_d_)|D_jk_).

If λ_e_< ${\hat{p}}_{jk}$ ≤λ_d_, then treat the next combination of patients at the same dose.

Note that in step 2, if two doses have the same value of P(p_j'k’_ ε (λ_e_, λ_d_)|D_jk_) in AE or AD, then either of the two doses can be chosen either randomly or based on clinical considerations. If no dose combination exists in AE and AD due to being at the boundaries of the dose matrix, then the next cohort of patients is treated at the same dose level [14]. In the software of the BOIN Comb design, the value P(p_j'k’_ ε (λ_e_, λ_d_)|D_jk_) is translated into a desirability score. As a result, the implementation of the BOIN Comb design can be based on a standard BOIN decision table with an additional desirability score table (an example of such a table taken from Zhou et al. [1] is shown (Table 6)).

3. The previous step is repeated until the maximum pre-specified sample size N is reached or the trial is stopped due to excessive toxicity per the dose elimination rule.
4. At the end of the trial, the MTD is determined by first applying isotonic regression in two dimensions to the observed DLT rates to smooth these rates so that they are monotonically non-decreasing when one drug level is fixed at a certain dose, and then selecting the dose for which the smoothed/isotonic DLT rate is closest to the target DLT rate ϕ.

**Table 6:** Desirability score table for the BOIN Comb design with the target DLT probability of 0.3 [1].

Number of Patients	Number of DLTs	Desirability Score
0	0	6
3	0	7
3	1	11
3	2	5
3	≥3	E
6	0	3
6	1	13
6	2	16
6	3	10
6	≥4	E
9	0	2
9	1	9
9	2	17
9	3	18
9	4	12
9	≥5	E

“E”: eliminate current and higher doses.

#### 3.1.2 BOIN waterfall design to find an MTD contour

For many drug combination trials, it is of interest to find the MTD contour, a set of multiple MTDs rather than a single MTD [41,42]. The extended design to find the MTD contour is called the BOIN waterfall design. This design finds the MTD contour via a sequence of one-dimensional dose-finding tasks known as subtrials. The subtrials are completed in order from the top to the bottom of the two-dimensional matrix formed by doses of one drug on each axis assuming a two drug combination. The DLT rate at each dose level is estimated based on the DLT data from all the subtrials using isotonic regression in two dimensions. For each row of the two-dimensional matrix, the MTD selected is the dose combination that has the smoothed DLT rate after isotonic regression closest to the target DLT rate ϕ. If all combinations in the row are overly toxic, no MTD is selected. The sample size required for finding the MTD contour is larger than that required for combination trials that aim to find a single MTD [41,42].

##### 3.1.2.1 Software

Software to implement the combination BOIN design to determine a single MTD or the MTD contour is available at www.trialdesign.org. The R package BOIN can also be used to implement the combination BOIN design [14].

##### 3.1.2.2 Advantages/limitations

As drug combination trials are becoming increasingly common, these designs are increasing in importance and use. The combination BOIN designs are easy to understand and implement and have comparable performance characteristics to model-based designs such as the partial ordering CRM and copula-type regression method [29].

The combination BOIN designs consider only toxicity and not efficacy in dosing decisions, which is important for immuno-oncology drugs where efficacy does not always increase with an increase in dose. They do not consider late-onset toxicities or responses.

## 4 Discussion

BOIN designs are a class of model-assisted dose-finding designs used in oncology trials, with the main aim being to estimate either the MTD or the OBD of a study drug. In BOIN designs that only consider the drug toxicity, the goal is to determine the MTD. In BOIN designs that consider both drug efficacy and toxicity, the goal is to determine the OBD, an optimal drug dose that is efficacious but not too toxic. In chemotherapy drugs, it is assumed in general that there is a monotone increasing dose-response relationship for both toxicity and efficacy. Thus, it is meaningful to find the maximum tolerated dose of the drug. However, in many of the newer immuno-oncology drugs, although the toxicity of the drug increases with an increase in dose, the efficacy of the drug does not always increase and could plateau at a lower dose. For such drugs, it is imperative to find the OBD, which is a dose that optimizes the risk-benefit trade-off. In this context, note that in the case of drugs where no DLTs are expected to be observed in the dose range being explored and where the drug is expected to be efficacious, pharmacodynamic/pharmacokinetic (PK/PD) guided escalation designs, and not BOIN designs, need to be considered to determine the drug dose to be used in further trials.

BOIN designs are relatively new and have seen rapid development not only in the methods literature but also in the frequency of their implementation in clinical trials; also the fit-for-purpose designation granted to the local BOIN design under the non-informative prior by the FDA emphasizes its importance and significance as a drug development tool. If the FDA extends the fit-for-purpose designation of the BOIN design to other BOIN designs such as TITE-BOIN and BOIN12 in the future, this would further emphasize the utility of this class of designs in drug development. BOIN designs provide a general framework to incorporate and investigate different aspects of dose finding, for example, toxicity, efficacy, binary or continuous outcomes, delayed toxicity and delayed efficacy and drug combinations. Thus, BOIN designs provide a complete suite of tools for dose finding in early phase trials, and a consistent way to explore different scenarios in a unified manner with easy access to software [1] for use to implement most of these designs; many of them including BOIN, TITE-BOIN, BOIN Comb have been implemented in real clinical trials, and even the very recent BOIN12 is currently being implemented in clinical trials. Further, in the BOIN designs, the dosing algorithm and dose selection (MTD/OBD) are independent in general, which is another advantage since methods other than isotonic regression, for example logistic regression, can be applied for dose selection. Although built upon Bayesian probability models, BOIN designs are generally operationally simple, have good statistical operating characteristics when compared to other dose-finding designs [5], and are also clinically sound especially when communicating with clinical investigators.

Due to the small sample size in early phase trials, BOIN designs do not consider patient heterogeneity, e.g., a mixture of patients who are sensitive and not sensitive to immune checkpoint inhibitors [21]. When the population of sensitive and non-sensitive patients can be pre-defined, BOIN designs may be used separately for each population. If this cannot be done and the sub-populations are unknown and need to be identified during the trial, a larger sample size is required and further research is needed as to how to apply BOIN designs in this case [21].

In summary, in this article, we have provided thumbnail sketches of the original BOIN design and its many extensions, discussing their advantages and limitations, software to implement them, examples of their use (Table 7 and Appendix) and the situation in which each of the designs in this class is suitable for use (Fig. 1).

**Table 7:** Summary of the BOIN Design and its Extensions.

Design	Description of Design	Advantages	Disadvantages	Software	References
BOIN	The BOIN design uses the observed DLT rate at each dose for determining the MTD of a new drug. This phase I design can be implemented in a simple manner, similar to that of the 3 + 3 design but has better operating characteristics, and has comparable or better operating characteristics than many model-based designs such as CRM, and model-assisted designs such as mTPI-2.	Although built upon Bayesian probability models, the BOIN design is operationally simple, since once λ_e_ and λ_d_ are pre-determined for the given target DLT rate, the dosing decision for each new cohort of patients is based mainly on comparing the observed DLT rate at the current dose with λ_e_ and λ_d_. The design selects the MTD more accurately and doses a larger percentage of patients at the MTD than the 3 + 3 design does, and it has a lower probability of overdosing patients than some other designs [10].	This design considers only toxicity in its dosing decisions without using efficacy data, which is an important limitation for immuno-oncology drugs where efficacy does not always increase with higher doses. It also does not consider late-onset toxicities or efficacy responses. BOIN mainly uses the data from the current dose level for dosing decisions and not data collected across all doses. However, it is a sequential design where the consecutive dosing decisions indirectly use the information from the adjacent doses, and this results in good statistical operating characteristics.	1) www.trialdesign.org 2) R package: BOIN 3) Desktop application: https://biostatistics.mdanderson.org/SoftwareDownload/SingleSoftware/Index/99	[5,10,11]
MT-BOIN	The MT-BOIN design(s) is an extension of the BOIN design that considers different toxicity types and grades.	MT-BOIN is simple to implement and has comparable operating characteristics to those of model-based designs such as MC-CRM. MT-BOIN is also more robust than model-based designs since it does not rely on a parametric dose-response assumption. In addition to non-nested and nested toxicities, MT-BOIN can handle drug combinations.	MT-BOIN does not consider late-onset toxicities or efficacy responses.	The boundaries of MT-BOIN are exactly the same as those in the standard BOIN design for non-nested outcomes. Thus, the BOIN software can be used to implement MT-BOIN.	[15]
gBOIN	The gBOIN design is a more general version of the BOIN design that can handle continuous, quasi-binary, and binary toxicity endpoints.	The gBOIN design has good statistical operating characteristics compared to existing designs that handle toxicity grades such as the quasi-CRM design [16]. It is simple to implement, since its dosing decisions involve comparing the sample mean of the endpoint with the two pre-specified boundaries of dose escalation and de-escalation, and do not involve any model fitting. It does not require a lead-in phase and its decision rules can be applied throughout the trial, unlike some model-based designs that need to collect preliminary data through a pre-phase before the model can be reliably estimated. Although gBOIN mainly uses data from the current dose level for dosing decisions, its performance is usually as good as or better than the model-based designs that borrow information across doses.	The required elicitation of weights (to determine a toxicity score) and a target involves a time-consuming collaboration between clinicians and biostatisticians. gBOIN does not consider late-onset toxicities or efficacy responses.	gBOIN can be implemented using the UnifiedDoseFinding R package available at https://cran.r-project.org/web/packages/UnifiedDoseFinding/index.html.	[16]
TITE-BOIN	In the TITE-BOIN design, new patients can be enrolled even when the DLT data are pending for some of the patients in the previous cohort. When there are no pending DLT data, it reduces to the BOIN design.	TITE-BOIN is easy to implement and is well-performing. It takes into account late-onset DLTs and rapid accrual, allowing dosing decisions even with pending DLT data from some patients in the current cohort. TITE-BOIN generally shortens the trial duration substantially compared to waiting for the occurrence of pending toxicity events. It is more flexible in choosing the target DLT rate and more accurate in MTD selection than the rolling 6 design. It has similar accuracy in MTD selection as TITE-CRM, but it has better overdose control and is easier to implement.	TITE-BOIN uses DLT data only at the current dose for dosing decisions, in contrast to TITE-CRM, which considers data from all dose levels. However, simulations show that the effect of using only the current dose data leads to negligible efficiency loss [25, 38]. TITE-BOIN assumes that the time to DLT is distributed uniformly over the DLT assessment window, similar to what TITE-CRM does. This design is robust to this uniformity assumption, but if reliable prior information is available on the distribution of the time to DLT, then an informative prior can be used to improve design efficiency [22]. TITE-BOIN does not consider efficacy endpoints for its dosing decisions.	www.trialdesign.org	[22,25]
TITE-gBOIN	The TITE-gBOIN is a non-parametric, model-assisted design that is an extension of the gBOIN design that accounts for toxicity grades based on both cumulative and pending numeric toxicity scores.	TITE-gBOIN is a robust design that is simple to implement. Simulations in various realistic scenarios show that TITE-gBOIN is comparable in performance to gBOIN and it has a higher probability of selecting the MTD correctly and allocating more patients to the MTD than other available approaches [26]. It reduces trial duration compared to designs that do not allow sequential dose allocation.	The design performance may depend on the appropriate specification of the quasi-Bernoulli endpoint such as the normalized ETS [26]. A time-consuming collaboration between clinicians and biostatisticians is required to accurately derive the quasi-Bernoulli endpoint utilizing the weight for each toxicity grade and the drug-specific toxicity profiles [26]. TITE-gBOIN does not consider efficacy responses.	Software to implement the TITE-gBOIN design is available at https://github.com/qingxiaa/titegboin.	[26]
BOIN-ET	BOIN-ET design, a phase I/II design, is an extension of the BOIN design that utilizes both binary efficacy and toxicity outcomes in determining the OBD	In general, the BOIN-ET design selects the OBD more accurately and puts a higher average number of patients at the OBD than the model-based TC and SHH designs [27]. It is simpler and easier to implement, is safer, and provides much better overdosing control than these designs [27].	When the efficacy rate is sufficient at lower doses, BOIN-ET tends to allocate more patients to doses higher than the OBD. BOIN-ET may not be suitable for solid tumors, where the RECIST criteria are used for the efficacy assessment; it may cause delays in implementing the dosing decisions because the RECIST efficacy evaluation usually occurs later than the toxicity evaluation. Hence, the design may be most applicable to trials where the response assessment period and the DLT assessment period are on similar time scales.	Takeda et al. provide SAS code upon request to implement the BOIN-ET design. The following reference provides example dosing decision tables for cohort sizes of 3 and 6 to implement the BOIN-ET design [27].	[27]
BOIN12	BOIN12 is a flexible phase I/II design that can be used to determine the OBD. Each cohort of patients is allocated to the dose that optimizes the toxicity-efficacy trade-off. While U-BOIN has 2 stages with only toxicity data being used in the first stage and both toxicity and efficacy data being used in the second stage, BOIN12 has only one stage and uses both categorical toxicity and efficacy data in this single stage.	BOIN12 is based on a utility trade-off function and is more general, while BOIN-ET is based on marginal toxicity and marginal efficacy rates and does not incorporate toxicity-efficacy trade-off in dose finding. The BOIN12 design is simple to implement, and it selects the OBD more accurately and doses more patients at the OBD compared to existing dose-finding designs such as the TC method, TEPI and 3 + 3 CE designs. The dosing decision table for the BOIN12 design can be used easily to make dosing decisions and allocate patients to a dose without any complex calculations [21].	In some immunotherapy trials, late onset toxicities and responses may be observed. This will preclude using the BOIN12 design, since it assumes that the toxicity and efficacy outcomes are available by the time of the dose assignment of the next cohort.	www.trialdesign.org	[21]
U-BOIN	U-BOIN is a utility-based, seamless Bayesian phase I/II design used to determine the OBD. The weights used in the utility function for different combinations of efficacy and toxicity (e.g. no response and no DLT, response and no DLT, no response and DLT and response and DLT) are chosen based on discussions with clinicians.	The U-BOIN design is simple to implement and is well-performing. It can be implemented in a trial using pre-determined decision tables and does not require complex model fitting and estimation [35]. Compared to a model-based design such as the TC method, U-BOIN identifies the OBD more accurately and is more robust [34]. Due to the incorporation of the first stage, U-BOIN also estimates the MTD accurately.	U-BOIN models efficacy and toxicity at each dose independently while model-based designs such as the TC method model efficacy and toxicity across all doses. Thus, there may be a potential efficiency loss for U-BOIN, although this loss is believed to be minimal or negligible [34]. U-BOIN is comparable to BOIN12 in accuracy of OBD selection, but it requires a relatively large sample size to guarantee a desirable performance due to the use of two stages. The sample size required by U-BOIN is usually greater than the sample size used in MTD-finding trials. In stage 2, U-BOIN assumes that both the toxicity and efficacy outcomes are available by the time of the dose assignment of the next cohort.	www.trialdesign.org	[34,35]
gBOIN-ET	The gBOIN-ET design is a phase I/II model-assisted, non-parametric design that is an extension of the BOIN-ET design and that accounts for ordinal graded efficacy and toxicity.	gBOIN-ET is simple and easy to implement in oncology trials than model-based approaches. Simulations investigating the operating characteristics of gBOIN-ET show that it has a higher performance than BOIN12, gBOIN, BOIN-ET in terms of the correct OBD selection, the average number of patients allocated to the OBDs, not selecting overdoses as the OBDs and not assigning patients to overdoses.	The design performance may depend on the appropriate specification of the quasi-Bernoulli endpoints. A time-consuming collaboration between clinicians and biostatisticians is required to accurately derive the quasi-Bernoulli endpoints utilizing the weight for each toxicity grade and efficacy grade. gBOIN-ET may select lower doses as OBDs if the low quasi-Bernoulli efficacy probability is mis-specified as the target quasi-Bernoulli efficacy probability. gBOIN-ET does not consider the accrual rate, the outcome evaluation period and the late-onset outcomes [36].	SAS code is available to implement the gBOIN-ET design.	[36]
TITE-BOIN-ET	This model-assisted design is an extension of the BOIN-ET design that considers both pending efficacy and toxicity data in the dosing decisions.	The design is robust, much simpler, and easier to implement than model-based approaches. TITE-BOIN-ET selects the OBD more accurately and puts a higher average number of patients at the OBD than model-based approaches such as the design by Thall and Cook and that by Jin et al. [37]. The trial duration is significantly shortened when using TITE-BOIN-ET compared to using designs without sequential enrollment [37].	When the efficacy response rate is sufficient at lower doses, the TITE-BOIN-ET design tends to allocate more patients to doses higher than the OBD. If patient accrual is faster than the outcome evaluation, then the available information may still not be sufficient even if the pending data are considered. In such a case, suspension rules as in Refs. [22,25] may need to be considered to delay the dosing decisions until adequate information is available [37].	Takeda et al. provide SAS code upon request to implement the TITE-BOIN-ET design. The following reference provides an example dosing decision table for cohort size 3 to implement the TITE-BOIN-ET design [37].	[37]
TITE-BOIN12	TITE-BOIN12 design is a utility-based phase I/II design that deals with late-onset toxicities and responses and allows the study to proceed with dosing the next cohort of patients even in the presence of pending outcomes for toxicity or response for some patients. It reduces to the BOIN12 design when there are no pending outcomes for toxicity or response.	TITE-BOIN12 is a well-performing design that allows continuous accrual while still ensuring patient safety and accuracy of OBD identification. In most cases, it has better over-dose control and higher accuracy of OBD identification, than model-based designs such as the TC method [39]. TITE-BOIN12 accommodates different shapes of dose-efficacy curves compared to the TC method. It shortens the trial duration and incorporates risk-benefit trade-off with input on the utility values from clinicians [39].	TITE-BOIN12 assumes that the time to DLT and efficacy are distributed uniformly over the assessment window, while calculating the STFT, which is similar to what is assumed in the TITE-BOIN and TITE-CRM designs [39]. This design is robust to this assumption, similar to what is demonstrated for TITE-CRM [40] and for TITE-BOIN [22]. However, if reliable prior information is available on the distribution of the time to DLT or efficacy, an informative prior can be used for either or both to improve design efficiency. No decision table can be generated for TITE-BOIN12 prior to the trial conduct.	www.trialdesign.org	[39]
Combination BOIN	The combination BOIN design(s) is used to design phase I trials that investigate a combination of two drugs with multiple dose levels for each drug. These designs can be used to determine the MTD or the MTD contour for a combination of drugs.	As drug combination trials are becoming increasingly common, these designs are increasing in importance and use. The combination BOIN designs are easy to understand and implement and have comparable performance characteristics to model-based designs such as the partial ordering CRM and copula-type regression method [29].	The combination BOIN designs consider only toxicity and not efficacy in dosing decisions, which is important for immuno-oncology drugs where efficacy does not always increase with an increase in dose. They do not consider late-onset toxicities or responses.	www.trialdesign.org R package: BOIN	[14,29,41,42]

## Authorship

RA was involved in the conception and design of the article, ML was involved in the supervision and critical review and editing of the manuscript, RL, CH, YP and DL were involved in discussions on the content of the manuscript, and critical review and editing of the manuscript.

## Funding

Ruitao Lin's research was partially supported by grants from the 10.13039/100000054National Cancer Institute (5P30CA016672 and 1R01CA261978).

## Declaration of competing interest

The authors declare that they have no known competing financial interests or personal relationships that could have appeared to influence the work reported in this paper.

## Appendix of example trials where BOIN designs are used:

### Example of BOIN Design:

A phase I clinical trial that used the BOIN design is the trial for an aurora kinase inhibitor (AKI) as monotherapy, in combination with docetaxel in patients with advanced solid tumors [11]. The docetaxel was an IV at a fixed dosage of 75 mg/m^2^. Six dose levels (20, 40, 80, 150, 250, 350 mg) of the AKI were investigated, and the dose was administered on days 1, 8 and 15 of a 28-day cycle. 36 patients were treated and the cohort size was 3. The BOIN design was used for dose finding with a target DLT rate of 0.25. The operating characteristics of the BOIN design were investigated for protocol preparation, and 10000 simulations were carried out for each of four scenarios. The statistical operating characteristics of the BOIN design were further compared to those of the CRM, mTPI and CCD (cumulative cohort design) designs. The BOIN design yielded similar results for MTD selection percentage and average number of patients allocated to the MTD as these other designs but had a lower risk of overdosing patients.

### Example of TITE-BOIN Design:

In the reference [22], the example of a phase 1 trial with a target DLT rate of 0.2 and five dose levels is used to illustrate the TITE-BOIN design.

The DLT assessment window was 3 months, the accrual rate was 2 patients/month, the maximum sample size was 30 and the cohort size was 3.

1) The first three patients were treated at the lowest dose level. At day 60, there were no DLTs in any of the patients and all 3 patients had data pending at this time. Based on the dosing table, accrual was halted until the first two patients finished the assessment period without experiencing a DLT. They both did not experience a DLT.
2) Based on the TITE-BOIN design, the dose level was escalated and patients 4, 5 and 6, were administered dose level 2. Patient 4 experienced a DLT by the arrival of patient 7 on day 165. Patients 5 and 6 had finished only 1/3 and 1/6 of their follow-up at this time without experiencing a DLT.
3) Per the design, the dose level was de-escalated and patients 7, 8 and 9 were dosed at dose level 1. By day 230, 3 out of the 6 patients in dose level 1 had completed their DLT assessment with no DLTs.
4) Per design, the dose level was escalated and patients 10, 11 and 12 received dose level 2. When patient 13 was to be dosed on day 255, one of the 6 patients in dose level 2 had experienced a DLT.
5) Per design, patients 13, 14 and 15 were dosed at dose level 2. 9 patients had been treated at dose level 2 by day 300, with 1 DLT observed and 5 pending patients. The trial was then halted for 15 days to collect more DLT data. On day 315, patients 3, 4, 5, 6, 10 and 11 had completed their follow-up while patients 12, 13, 14, 15 had been followed for 75, 60, 45 and 30 days respectively, and the DLT assessment period was 90 days. The STFT was (75 + 60+45 + 30)/90 = 2.33 for dose level 2, which was greater than the escalation boundary of 2.15.
6) Per design, the dose level was escalated and patients 16, 17 and 18 were treated at dose level 3.
7) One DLT had been observed at dose level 3 before the arrival of patient 19. Per design, the dose level was de-escalated and patients 19, 20 and 21 were administered dose level 2.
8) At the end of the trial, dose level 2 was selected as the MTD. At dose level 2, 4 out of 21 patients had experienced a DLT, resulting in a DLT rate of 0.19.
9) The entire trial with the TITE-BOIN design took 615 days or 20.5 months, while it would have taken 1200 days or 40 months with a traditional design, which requires a complete DLT assessment for each cohort of patients (either experience a DLT or complete the DLT assessment period) before enrolling the next cohort of patients.

### Example of BOIN12 Design:

The implementation of the BOIN12 design is illustrated in the following reference using the methotrexate (MTX) plus erlotinib plus celecoxib trial [21]. There were 5 dose levels of MTX (3, 6, 9, 12 and 15 mg/m^2^). The MTX was given in combination with 150 mg erlotinib and 200 mg celecoxib. The sample size used was 30 and the cohort size was 3. The highest toxicity probability ϕ_T_ was assumed to be 0.35 and the lowest acceptable efficacy probability ϕ_E_ was assumed to be 0.25. The escalation and de-escalation boundaries corresponding to ϕ_T_ from the BOIN design were λ_e_ = 0.276 and λ_d_ = 0.419.

1) The first cohort of 3 patients were dosed at the lowest dose, dose level 1, i.e. 3 mg/m^2^ MTX. No patient experienced a DLT or response. Hence the observed toxicity rate of 0 was < λ_e_ of 0.276. Based on the dosing algorithm step 2 c) and the desirability score table (Table 3 in Lin et al. [21] – the desirability score of dose levels 1 and 2 were 35 and 60 respectively), the dose level was escalated since dose level 2 had a higher desirability score. The next cohort of 3 patients was dosed at dose level 2.
2) Among these 3 patients in dose level 2, one patient experienced a DLT and one experienced a response. Based on the dosing algorithm step 2 c) and the desirability score table (Table 3 in Lin et al. [21] – the desirability score of dose levels 1, 2 and 3 were 35, 44 and 60 respectively), the dose level was escalated since dose level 3 had the highest desirability score. The next cohort of 3 patients was dosed at dose level 3.
3) The next cohorts of patients were dosed similarly by continuously updating the desirability of the doses and making the dosing decisions accordingly until the maximum sample size of 30 was reached.
4) The number of patients dosed, patients with DLTs and responses in the 5 doses at the end of the trial were (3, 3, 6, 12, 6), (0, 1, 1, 3, 3) and (0, 1, 2, 6, 3) respectively. The final estimates of the utilities of the five doses were 40, 46.7, 53.3, 60 and 50 respectively.
5) Thus, dose level 4 (12 mg/m^2^ MTX) with the highest utility value was selected as the OBD.

### Example of TITE-BOIN-ET Design:

To illustrate the use of the TITE-BOIN-ET design [37], considered a phase I/II study of gilteritinib to assess the safety, tolerability and pharmacokinetic effects of gilteritinib in FLT3 mutation-positive (FKLT3mut+) AML patients. A total of 252 patients with AML received a once-daily dose of oral gilteritinib in one of seven dose escalation cohorts (n = 23; doses - 20, 40, 80, 120, 200, 300 and 450 mg) or dose expansion cohorts (n = 229). The dose finding was based on an accelerated titration design and safety was monitored using the Bayesian logistic model [37]. The patient enrollment was 22 months, the DLT observation period was 30 days for the dose escalation cohorts and the median time to best response for FLT3mut + patients at dosages ≥80 mg was 7.2 weeks. From the trial, the number of patients who had DLTs and the number who achieved composite remission (CRc) was known. The MTD was established to be 300 mg and the dose chosen as the starting dose for later studies was 120 mg.

The trial was re-analyzed using the TITE-BOIN-ET design. Based on the results of the trial, the following parameters were assumed for the simulations of the TITE-BOIN-ET design. The DLT observation period was assumed to be 30 days, the efficacy assessment period to be 60 days and the accrual rate to be 1 patient per 2.6 days. A bell-shape curve was assumed for the true underlying probability for CRc and a monotonic pattern was assumed for the true probability of DLT, based on the observed rates in the trial. The target DLT rate was assumed to be ϕ = 0.3 and the target efficacy rate to be δ = 0.6. The design parameters used were ϕ_1_ = 0.1ϕ, ϕ_2_ = 1.4ϕ and δ_1_ = 0.6δ, leading to optimal values of λ_e_, λ_d_ and η_1_ of 0.14, 0.35, and 0.48. The simulations were done for sample sizes of 48, 96, 144 and 252, the cohort size was 3 and the starting dose was 20 mg. The simulations showed that the TITE-BOIN-ET design accurately selected the OBD and shortened the trial duration compared to the original trial design.

### Example of Combination BOIN Designs:

To illustrate the combination BOIN designs, The reference by Lin and Yin [29] considers an already completed trial with four doses of neratinib (120, 160, 200 and 240 mg) and 4 doses of temsirolimus (15, 25, 50 and 75 mg). The trial used the modified non-parametric up-down method for dose-finding decisions. The MTDs were defined to be the doses whose estimated toxicity rates were closest to but lower than the target toxicity rate of 0.33.

The authors used the combination BOIN design to redesign the trial, by setting ϕ_1_ = 0.6ϕ and ϕ_2_ = 1.4ϕ, leading to an optimal interval of (0.260,0.395). The maximum sample size used was 60 and the cohort size used was 2; the first cohort of patients was treated at the lowest dose of neratinib and temsilorimus. Implementing the combination BOIN design, the authors obtained the estimated toxicity probability at each dose level at the end of the trial. The dose combination with estimated DLT closest to but lower than 0.33 was selected as the MTD. The combination BOIN design was shown to quickly find the MTD and dose a large percentage of patients at the MTD.

Simulations were also conducted by Lin and Yin [29] to determine multiple MTDs (MTD contour). The maximum sample size used was 60 and the cohort size used was 1 or 2. 1000 simulations were conducted and 2 MTDs were chosen at the end of each simulation. Each simulation was started from dose combinations (1, 2) and (2,1). The simulation results included the percentages of selections of the MTDs and overly toxic dose combinations and the corresponding percentages of patient allocations [29]. In comparison to the combination BOIN design that chose a single MTD, the combination BOIN design that chose 2 MTDs 1) resulted in a higher percentage of MTD selections, 2) assigned fewer patients to the MTDs, and 3) allocated as well as selected a larger number of overly toxic dose combinations.

## Footnotes

1 BOIN has been used in a variety of oncology trials including those for pediatric tumors (ClinicalTrials.gov identifier: NCT02354547), adult tumors (ClinicalTrials.gov identifiers: NCT03577704, NCT0302316, NCT02942264, NCT03318900, NCT03600155, NCT0205075, NCT03740256, NCT03330028, NCT03114462, NCT03036904, NCT02705196, NCT02942095, NCT03740256, NCT03784677, and NCT03760081), solid tumors (breast [ClinicalTrials.gov identifier: NCT0302316], brain [ClinicalTrials.gov identifier: NCT02942264], ovarian [ClinicalTrials.gov identifier: NCT03318900], stomach [ClinicalTrials.gov identifier: NCT03330028], neck [ClinicalTrials.gov identifier: NCT03114462], lung [ClinicalTrials.gov identifier: NCT02942095), bladder [ClinicalTrials.gov identifier: NCT03740256], prostate [ClinicalTrials.gov identifier: NCT03784677], and germ cell [ClinicalTrials.gov identifier: NCT03760081]), and liquid tumors (leukemia [ClinicalTrials.gov identifier: NCT03600155], and lymphoma NCT03114462]). BOIN has been used for various treatment agents, including chemotherapy (ClinicalTrials.gov identifier:NCT02942264), radiotherapy (ClinicalTrials.gov identifier: NCT03114462), checkpoint inhibitor (ClinicalTrials.gov identifier: NCT03600155), monoclonal antibody (ClinicalTrials.gov identifier: NCT03577704), oncolytic virus (ClinicalTrials.gov identifier: NCT02705196), and T-cell immunotherapy (ClinicalTrials.gov identifier: NCT03318900). BOIN also has also been used in non-oncology trials, such as stem cell therapy for stroke patients.

2 Based on the FDA's review of the local BOIN design under the non-informative prior for the fit-for-purpose designation, an erratum to the original de-escalation and stay at the same dose rules of this design [11] was published recently. The de-escalation rule was changed from ${\hat{p}}_{j}$ ≥λ_d_ to ${\hat{p}}_{j}$ >λ_d_ and the stay at the same dose rule was changed from λ_e_< ${\hat{p}}_{j}$ <λ_d_ to λ_e_< ${\hat{p}}_{j}$ ≤λ_d_. This does not affect the design's application to an actual trial as it is virtually impossible to meet the exact boundary criteria in practice (Erratum to Liu and Yuan [11], 2022).

3 The relationship between the two toxicity outcomes can be nested or non-nested, depending on whether one toxicity outcome is a subset of the other outcome or not. For example, if one toxicity outcome is defined as grade ≥ 3 toxicity in the liver and the second toxicity outcome is grade ≥ 3 toxicity in other organs (not liver), then the two outcomes are non-nested. When the two toxicity outcomes are defined based on the maximum toxicity grade, say the first toxicity outcome is the event of maximum toxicity grade ≥ 2 and the second outcome is the event of maximum toxicity grade ≥ 3, then the second toxicity outcome is nested in the first toxicity outcome.

4 Computed as the arithmetic sum of different grades and types of toxicity, weighted by the severity weights that are elicited from clinicians [16].

5 Computed by using a weighted sum, where the severity weights were estimated via regression using historical data [16].

6 Computed as the Euclidean norm of the severity weights [16].

7 Defined as quantitative measurement of the overall toxicity severity for each patient, normalized to a value between 0 and 1 [20].

8 Setting u_3_≥u_2_ means that the patient is willing to tolerate toxicity in exchange for efficacy and generally leads to a more thorough exploration of the dose space compared to setting u_3_<u_2_ [21].
